# Building the English health visitor workforce as a result of the Health Visitor Implementation Plan 2011–2015: a survey study of career progression and retention for newly qualified health visitors

**DOI:** 10.1017/S1463423619000604

**Published:** 2019-09-09

**Authors:** Judy Brook, Valerie Thurtle, Joy Murray

**Affiliations:** 1 Senior Lecturer, Division of Health Services Research and Management, School of Health Sciences, City, University of London, London, UK; 2 Senior Lecturer, Division of Health Services Research and Management, School of Health Sciences, City, University of London, London, UK; 3 Senior Lecturer, Department of Health & Social Sciences, University of the West of England Bristol, Bristol, UK

**Keywords:** health visitor, implementation plan, public health, retention, survey, workforce

## Abstract

**Aim:**

This study aimed to explore the extent to which health visitors who trained and qualified in both Greater London and the South West of England between September 2011 and January 2016 were employed in health visiting posts and have remained in the profession.

**Background:**

In 2011, the UK Government launched the Health Visitor Implementation Plan ‘A Call to Action’ (Department of Health, 2011) to develop the health visitor workforce by training 4200 health visitors over a four-year period. By April 2015, 4000 additional health visitors were trained, but the total workforce has since fallen back to pre-Implementation Plan size.

**Methods:**

Data were collected using a survey, completed online by participants. All participants had undertaken a health visitor education programme at one of two participating universities. The survey was distributed in January 2017 and completed by 180 individuals. Quantitative data were analysed using SPSS; association was assessed using individual chi-square tests or Fisher’s exact test. Free-text responses were thematically analysed.

**Findings:**

Most (153; 87%) participants were still working as health visitors. Length of time spent working in the community prior to completing health visitor training was associated with staying in the role ( *χ*^2^ (with Fisher’s exact test = 7.998, *P* = .027). Current pay was associated with attrition from the health visitor workforce ( *χ*^2^ (with Fisher’s exact test) = 67.559, *P* < .001.). The majority who had left the health visitor role were on higher pay bands in their new post compared to those that had stayed (12; 60%). Bronfenbrenner’s (1979) theory of socio-ecological development was used as a framework to interpret the results. While participants made an active choice to join the profession, leaving was influenced more by factors outside their control. To influence health visitor retention, both local and strategic changes are required.

## Introduction

A strong health workforce is highlighted by World Health Organization (2017) as one of the building blocks of an effective health system. With increased focus on international and national policies to support population health, development and sustainability of the public health workforce in particular is a global imperative (Zodpey *et al.*, 2018). Even in high-income countries with well-established healthcare systems, there are persistent shortages of public health practitioners; in the United States, public health nurses make up 16% of the total public health workforce and are the largest single group (Beck *et al.*, 2014) but a deficit has been well-documented (Beck and Boulton, 2016). Competition between private healthcare organisations and governmental public health agencies have impacted on recruitment (Yeager and Wisniewski, 2017), highlighting the need for greater understanding of the influences on public health nurse retention.

In England, the public health nursing workforce consists of health visitors (HVs), school nurses and occupational health nurses. HVs work with pre-school children and families to promote healthy lifestyles and prevent illness. They are nurses or midwives who have undertaken a further year of study to gain a specialist public health qualification. They make up approximately 1.7% of the English nursing workforce (Department of Health (DH), 2015), with just over 8000 whole-time equivalent HVs in post in December 2017 (NHS Digital, 2018).

## Background

In 2011, the UK Government launched a significant policy initiative to develop the HV workforce. The Health Visitor Implementation Plan ‘A Call to Action’ (DH, 2011) set out three integrated work streams: growing the workforce, professional mobilisation and aligning delivery systems. These areas for development were intended to collectively strengthen the health visiting service over a four-year period and led to unprecedented student numbers in the one-year educational programmes in higher education institutes that equip nurses and midwives to become HVs, whilst innovative practice and new service models influenced the way both established and newly qualified HVs worked.

Investment in HV education and recruitment was based on the premise that newly qualified practitioners would enter the profession by taking up health visiting posts and therefore contribute to the public health agenda in England. However, investment was part of a complex picture of UK National Health Service (NHS) transformation and economic reform including radical changes to the structure of the NHS and public health services.

In April 2015, a Department of Health position statement (DH, 2015) indicated that the workforce had been successfully increased by 4000 new HVs. Although this announcement was met with optimism (Bennett, 2015), the structural changes introduced uncertainty around the future of both HVs’ roles and job security (Unite the Union, 2016) and presented a diverse picture for newly qualified HVs in relation to employment terms and conditions. Since 2015 the number of HVs in England has fallen by 19% and the HV workforce is now back to the pre-Implementation Plan size of 8000 whole-time equivalent HVs (NHS Digital, 2018).

For such policy and investment to successfully support the needs of children and young people, workforce development must be sustainable, which requires understanding of why nurses and midwives choose to become HVs and how they can be retained in post (Whittaker *et al.*, 2013). The changing nature of the workforce, with an estimated 50% now newly qualified (Centre for Workforce Intelligence, 2012) presents challenges in terms of the established leadership experience and support within health visiting services. The UK Government has invested extensive public funds; research to establish the impact of this investment and how the regeneration can be sustained is important to inform the future recruitment and retention of practitioners in this area of public health practice.

## Theoretical framework

Previous research indicates that the career choices nurses and midwives make are influenced by a wide range of contextual factors (Hickey *et al.*, 2012; van Iersel *et al.*, 2016). Bronfenbrenner’s (1979) theory of socio-ecological development offers insight into not just the context but also the complexity of interactions that influence the individual situation. Bronfenbrenner (1979) stressed the importance of the context of multiple environments on the way people develop and grow. He identified five ecosystems that exert external influence, inevitably interact and influence every aspect of an individual’s life. Hickey *et al.* (2012; 2013) premise that career choices are a product of the interactions between the individual and their environment and suggest that the nursing student or graduate sits at the centre of Bronfenbrenner’s concentric (or ‘nested’) system of levels of influence. The microsystem takes account of their pattern of interpersonal relationships, experiences, personal characteristics and intrinsic motivational factors. The mesosystem interrelates the microsystems of family, neighbourhood setting and friendships. The exosystem takes account of wider events in their clinical practice setting and the macrosystem addresses overriding policy or beliefs that may affect their career choices. The influence of time on the development of career choices is illustrated by the chronosystem, which recognises that choices are not fixed and are developed through a period of transition.

In order to become a HV, nurses and midwives must make a conscious choice to undertake the educational programme and then continue their journey within the workforce. Drawing on the work of Hickey *et al.* (2012; 2013), the five systems of Bronfenbrenner’s Ecological Systems Theory have been employed as the lens through which to view the findings and explore the nuance of context within which the participants made their career choices.

This cross-sectional survey design study aimed to explore the extent to which HVs who trained and qualified in both Greater London and the South West of England during the Health Visitor Implementation Plan were employed in health visiting posts and have remained in the profession.

The objectives were:

1. To identify the workforce destination of newly qualified HVs, educated at two universities in the geographical areas in question, at the time of data collection.
2. To identify the number who had taken up roles in other areas of nursing or midwifery, or outside the profession.
3. To explore the reasons why newly qualified HVs decided not to work in the HV role.
4. To explore the relationship between characteristics of the newly qualified HVs and retention and progression in the health visiting workforce.
5. To derive conclusions and recommendations regarding the retention of newly qualified HVs from the findings of the research.

## Methods

### Sample

All students who completed the HV education programme at two UK Universities, one in the West of England and one in Greater London from September 2011 to January 2016, were invited to participate in the study. These universities were chosen because they covered diverse demographical areas, offering an opportunity to explore differences and similarities in career journey linked to defined variables. The potential sample for the study was 150 alumni from the Greater London University and 800 from the South West University Public Health Nursing Programme (health visiting). This represented 19% of the national total of newly qualified HVs.

Participants were recruited using purposive sampling techniques and all participants were volunteers. Inclusion criteria were that potential participants had undertaken the HV education programme at one of the participating universities between September 2011 and January 2016. Participants who started but did not complete the HV education programme were excluded, as were any students who, at the time of data collection, were studying on education programmes that were linked to the public health team. Purposive sampling ensured that the opinions and experiences of a range of participants from both universities, in different clinical roles and geographical areas were heard.

### Recruitment

The Alumni Departments emailed all registered alumni that met the inclusion criteria, with details of the study, an information sheet and a link to the survey. The study was also advertised via social media. The emails were predominantly sent to personal addresses of the participants, designed to mitigate bias towards those who had stayed in the profession. Completion of the survey, including questions to confirm their understanding of the terms outlined, was taken as consent to participate.

### Survey design and data collection

The HV alumni data were collected using an online survey. A survey was chosen as a cost-effective means of gathering data from a geographically disparate population of alumni, due to its speed of completion, convenience for the respondent and low administrative costs (Evans and Mathur, 2005). Although non-response bias is recognised as a limitation of survey data collection (Cho *et al.*, 2013), the challenge of physical location prohibited focus groups or face-to-face interviews. The survey was electronic rather than paper based as this was likely to be more accessible to potential participants. The survey consisted of a maximum of 27 questions to elicit quantitative and free-text data around individual characteristics. Required responses were either yes/no, choice from a list or free-text answers. Questions were devised to collect data aligned to the study objectives and were informed by contemporary literature focusing on the Health Visitor Implementation Plan and workforce development. Questions asked about initial Nursing and Midwifery Council (NMC) registration, career choices, level of academic achievement, motivations and influences whilst undertaking the HV education, and employing organisation, geographical placement location. The survey was piloted within the participating organisations to determine question clarity and eliminate ambiguity and was distributed to the study population in January 2017.

### Data analysis

The quantitative data were analysed using SPSS software. Ethnicity, previous role, reasons for leaving and reasons for wanting to be an health visitor were grouped into broad themes, so that frequencies could be computed. Frequencies were calculated and association between predictor variables and the outcome variable (retention in health visiting workforce) was assessed using individual chi-square tests. Where expected frequencies were below 5, Fisher’s exact test was used.

Two free-text questions were asked in the survey about motivations for becoming a HV (all participants) and for leaving the profession (limited to participants who were leavers only). Data from these survey responses were collated and independently categorised by two members of the research team, to increase objectivity. The categories were then compared and a final set of themes agreed in order to facilitate useful insight into the motivations of HVs who qualified in both geographical areas.

### Ethical considerations

The study was approved by University Research Ethics Committee in May 2016 (Staff/16-17/03).

The three researchers on the study were lecturers at the universities, so the independence of the research and any conflicts of interest were set out in the participant information sheet. The questionnaire was returned anonymously, which mitigated coercion, conflict of interest or partiality. All participation was voluntary and the participants could opt-out prior to submitting the survey should they not want to engage or feel overexposed to research.

## Findings

### Survey

The survey was completed by 180 individuals, yielding 160 complete sets of data. Data were included from 17 out of the 20 incomplete survey responses. Seventy-three percent of responses were from alumni who belonged to the university in the south west of England (127) and 26% (46) from the Greater London university, with seven participants not answering this question.

### Demographics

The largest group of participants were aged 36–40 (38; 21%), female (177; 98%) and described themselves as White British (116; 64%), despite the wide ethnic variation in the participants from Greater London, including Black African/Caribbean, Asian/Bangladeshi and mixed. A large number of participants (87; 59%) held an adult nursing registration, with 44 (25%) registered as children’s nurses, 19 (11%) mental health nurses and 24 (13%) midwives. The majority (72; 40%) had more than 10 years of experience before undertaking the public health nursing programme; only 9 (5%) were newly qualified nurses or midwives. Participants left a wide range of previous roles to undertake the public health nursing programme, though the majority (*n* = 106, 58.9%) had previously worked in the community before undertaking the programme, over half for more than three years.

More participants (54.9%) undertook the education programme at undergraduate bachelors (BSc) as opposed to postgraduate diploma (PGdip) level.

## Quantitative results

### Retention in the workforce

Most (153; 87%) participants who qualified were still working as HVs at time of data collection and the majority (101; 58%) were still working for the organisation that supported them through the programme. Twenty (11%) of the participants had progressed to become specialist HVs or managers. The majority of those still working as HVs was paid at the national agreed pay rate (Agenda for Change band 6) (136; 89%), and 16 (10%) were paid at a higher level (Agenda for change band 7).

Twenty-two (12%) participants were no longer working in an HV role. Most participants left health visiting (12, 55%) after one to two years. These individuals were employed in a range of roles, including non-NHS roles (3; 9%), not working (3; 14%) and back into nursing or midwifery (11; 50%). Fourteen (64%) of those who left health visiting would consider returning. The variables’ relationship with retention in the health visiting workforce was calculated.

### Community experience

**Figure 1.**
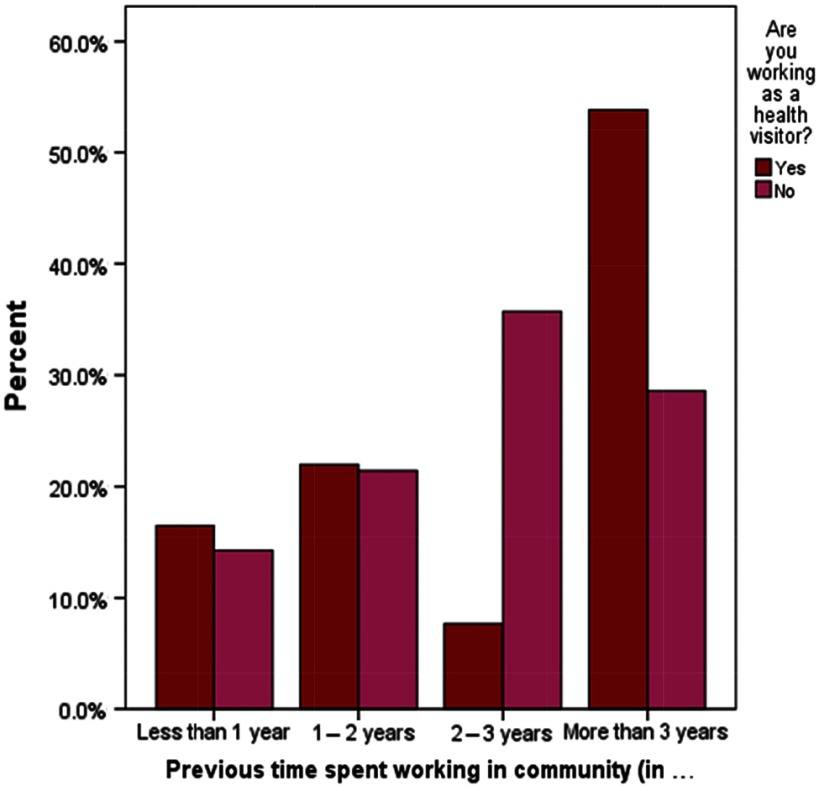
Time spent working in the community in years by health visitor retention status

Whilst previous community experience was not associated with retention in the HV workforce ( *χ* ^2^ = .139, *P* = .449), length of time spent working in the community was associated with whether participants were still practising as an HV ( *χ* ^2^(with Fisher’s exact test = 7.998, *P* = .027). The majority of the participants (49; 54%) still working as HVs had more than three years of work experience in the community (Table 1, Figure 1).

**Table 1.** Percentages of previous community experience in years, by health visitor retention status

	Are you still a health visitor?	
	Yes (*n* = 153)	No (*n* = 22)
Less than one year	24 (16%)	3 (14%)
One to two years	33 (22%)	5 (21%)
—Two to three years	14 (8%)	8 (36%)
More than three years	82 (54%)	6 (29%)

### Remuneration

**Figure 2.**
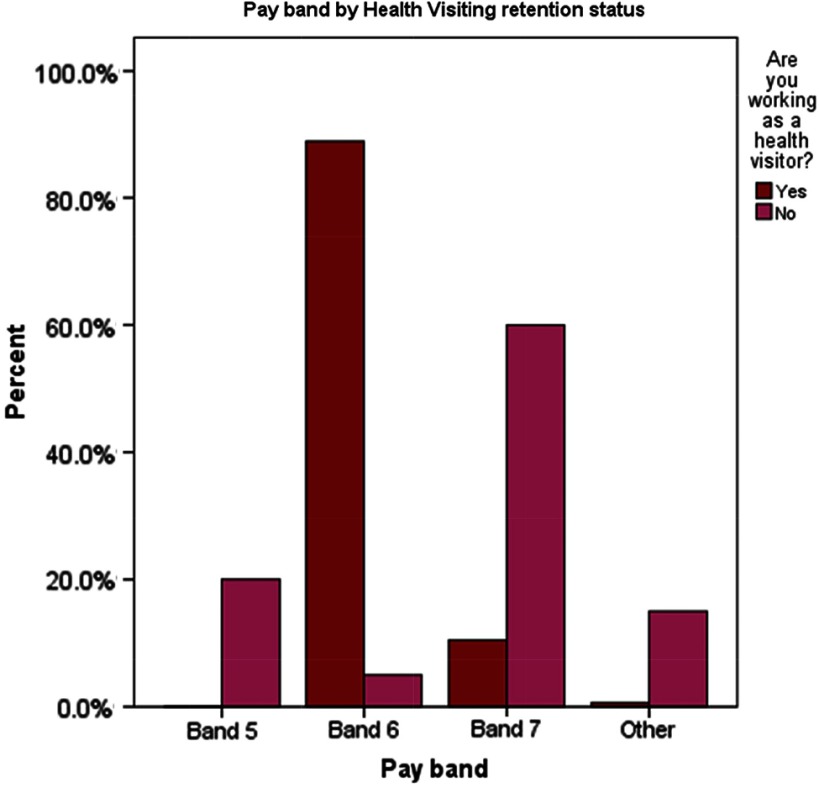
Pay band by health visiting retention status

Current pay was associated with retention in the HV workforce ( *χ* ^2^(with Fisher’s exact test) = 67.559, *P* < .001.) The majority of individuals who were still HVs were on pay band 6 (136; 89%), whereas the majority of those who had left were on pay band 7 in their new post (12; 60%) (Table 2, Figure 2)**.**

**Table 2.** Pay band by health visiting retention status

	Still working as a health visitor	
Pay band	Yes	No
Band 5	0	4 (20%)
Band 6	136 (89%)	1 (5%)
Band 7	16 (10%)	12 (60%)
Other	1 (1)	3 (15%)

### Professional variables

No association was found between retention in the HV workforce and type of UK NMC registration prior to undertaking the HV education programme (eg, adult nurse, children’s nurse, mental health nurse, midwife) ( *χ* ^2^ (with Fisher’s exact test) = 3.912, *P* = .481). The health organisation supporting the student through the programme ( *χ* ^2^(with Fisher’s exact test) = 24.876, *P* = .226) was not associated with retention in the workforce, nor whether participants had to relocate to complete their HV programme ( *χ* ^2^ = 2.742, *P* = .150).

### Academic variables

The university at which students completed their degree had a non-significant association with retention in the HV workforce ( *χ* ^2^ = 3.566, *P* = .068); however, the level at which the degree was completed was not associated with retention in the HV workforce ( *χ* ^2^ = .898, *P* = .368) and neither was academic achievement when completing the programme at BSc ( *χ* ^2^ = .898, *P* = .368) or postgraduate level [ *χ* ^2^ (with Fisher’s exact test) = 2.043, *P* = .440].

## Results from free-text answers

### Motivations for becoming an HV

Participants were asked why they had wanted to be an HV and 160 participants responded. Their responses were grouped in to categories and are outlined in Table 3. The dominant reason given was to work with families (*n* = 56), followed by an interest in public health, health promotion and preventative work (*n* = 36) or taking the opportunity for career progression and development (*n* = 23).

‘*I always was interested in child development and wanted to work with children after working many years with adults. A refreshing change*.’ (Response 3)

‘*Working with families for a longer period of time, using public health knowledge and skills in another area of practice and expand my own knowledge and skills*.’ (Response 108)

**Table 3.** Motivation for becoming a health visitor

Reason given for becoming a health visitor	Number of participants
Wanted to work with children and families	56
Interest in public health, health promotion and prevention of illness	36
Better working conditions such as work pattern and pay	26
Opportunity for career progression, professional growth and development	23
To work in the community	17
To make a difference or have an impact on people’s lives	16
Was inspired by own experience of HV	11
To use existing skill set effectively	10
Wanted a change	8
To implement early intervention	5
To provide continuity of care	3
Wanted to leave previous role	2
To work for the family nurse partnership	1

Twenty-four participants were motivated to become an HV because of the perceived improvement in working conditions such as Monday to Friday working and improved pay.

‘*Wanted Christmas and weekends off, set hours of work (no shifts). Liked the role, wanted to work with families over longer periods, build relationships with them*.’ (Response 19)

In addition, 11 participants highlighted their own experience of a particular HV either as a parent themselves or while taking part in an HV placement as a pre-registration nurse. Becoming an HV in these cases often fulfilled a long-held aspiration.

‘*As a student nurse in 1985 on a health visiting placement I was struck by the importance of health education, prevention and support as early as possible in client lives. The Implementation Plan gave me the opportunity to fulfil a long term ambition to qualify as a health visitor*.’ (Response 90)

Ten participants saw the move to health visiting as a way of utilising their existing skill set effectively and recognised that though they would be developing new skills, they could draw on established ability in communication, working with children or neonates, or health promotion in their new role.

### Motivations for leaving the profession

The reasons why HVs left the profession and the attraction of their new chosen roles were categorised (outlined in Tables 4 and 5) and were dominated by factors associated with the practice environment (*n* = 10) and inability to progress.

‘*Culture of blame, bullying, little support, unrealistically high expectations of new staff, training not valued, no preceptorship = burnout*.’ (Response 23)

**Table 4.** Reasons for leaving health visiting

Reason for leaving health visiting	Number of participants
Workplace culture (including discrimination, poor support, bullying/workplace structure including training, poor funding, service restrictions)	10 (not restricted to one employing organisation)
To further career/pursue other interests	7
Participant relocation	3
Pay and/or poor clarity over pay bands	2
Inability to get a job as a health visitor (HV) despite wanting and trying to	2
Lack of ability to progress	2
Health concerns	2

**Table 5.** Reasons for participants’ new choice in career

Reasons for participants’ new choice in career	Number of participants
Increased salary, improved work prospects and work environment	6
A genuine interest in the area	6
Feeling able to use skills and make a difference through more client contact	5
Autonomy and flexibility	4
Feeling respected and valued	2
Not being in the UK National Health Service (NHS) or health visiting	1
Teaching opportunities	1
Inability to find health visitor (HV) role despite wanting to	1

Five participants left health visiting because of influences in their personal lives, for example, family factors such as accompanying their husband working abroad and seven left to pursue other interests, including the opportunity to work in higher education.

The move to new roles was influenced by a variety of factors but increased salary, opportunities to progress and perceived benefits to the work environment were cited by six participants. Six participants also felt a genuine interest in their new chosen area of work.

*‘The salary, the opportunity and my new manager’s belief in me and respect for me*.’ (Response 20)

*‘An area of interest and gives me more freedom to develop services*.’ (Response 34)

Ultimately the reasons for leaving and taking up new roles were multi-faceted and involved a combination of ‘push’ factors (eg, dissatisfaction with current situation) and ‘pull’ factors (eg, appeal of new employment).

## Discussion

This study aimed to explore the extent to which HVs who trained and qualified in two geographically distinct areas of England between 2012 and 2016 were employed in health visiting posts and have remained in the profession. The results of the online survey offered insight into the varying characteristics of the newly qualified HVs and retention and progression in the health visiting workforce.

The statistical analysis of the survey data identified two significant associations: between working in the community for more than three years prior to choosing to undertake the public health nursing programme and remaining in the profession; and leaving health visiting to work in a higher paid role. The lack of statistically significant association between other factors was equally interesting as these results may serve to dispel myths that have perpetuated anecdotally, for example, that more academically able students leave the profession or prior community experience is essential. The free-text responses to the survey questions provide additional insight into motivations for moving into and out of the health visiting profession and may help to inform recommendations for policy and practice.

Bronfenbrenner’s Ecological Systems Theory offers the potential to gain insight into where interventions may be most effectively placed to retain the workforce. Bronfenbrenner (1979) places the individual at the centre of five ecosystems, which suggests that career decisions are influenced by a constellation of contextual factors, the interrelationships between them and personal characteristics and experiences (Hickey *et al.*, 2012; 2013). Participant responses in this study reinforced this premise; they were active agents in their move to the new profession and these decisions were influenced by their interpretation of the situation, in turn formed by their own life experience and attributes. Inspiration from their experiences with their own HV motivated some participants to join the profession, whilst others cited a personal desire to make a difference, to participate in early intervention, be involved in public health or to use an existing skill set in a new environment. Reasons cited for leaving health visiting that pertained to the individual were limited to personal health concerns or wanting to further their career. This suggests that while participants made an active choice to join the profession, decisions to leave were influenced to a greater extent by factors outside of their control.

Bronfenbrenner identifies the microsystems within which an individual exists, for example, their work, family and peers, and the links between these (the mesosystem) as influences on decision making and development. Participants cited the work environment, workplace culture and work–life balance as both motivators to join the profession and reasons for leaving, which may suggest a gap between expectation and the reality of the role. This may also relate to the finding that the length of time spent in the community prior to becoming an HV was significantly associated with retention. Those HVs who had realistic expectations about working in the community, established through three or more years of prior experience, may be less likely to suffer disappointment with the work environment. There are opportunities to influence perceptions of community working through clinical placements during pre-registration nursing or midwifery programmes, and student nurses often have strong views about the community as a work environment. It may be perceived as less attractive due to the lack of requirement for technical skill and high workloads (van Iersel *et al.*, 2016), potentially because students find it difficult to recognise the complexity of community care. Conversely, it is perceived positively if their placement experiences were inspiring, indicating that preference for work settings tends to be established early, that can remain unchanged (Murphy *et al.*, 2012; Peters *et al.*, 2015) and that the quality of these experiences will influence post-registration career choices (Baglin and Rugg, 2010).

Perceived characteristics of the HV role, such as better working conditions (Monday to Friday working, lack of shifts) and opportunities for career progression and community working, impact on relationships between work and family (the mesosystem). The practice environment has been identified in both nursing and health visiting literature as influential in decisions to stay or leave. Low job satisfaction, few opportunities for development and experiencing work–family conflict are cited as key factors (Flinkman *et al.*, 2010; Whittaker *et al.*, 2013; Twigg and McCullough, 2014). Work–family conflict is most acute at the beginning of a career and between 30 and 40 years of age (Simon *et al.*, 2004), which aligns with the demographics of this study sample. Work–family conflict occurs when emotions and behaviours in the work domain spill over into the family domain with negative effects (Edwards and Rothbard, 2000), often associated with a decision to leave (Battistelli *et al.*, 2012). Health visiting has been identified as a role that requires emotional labour (Taylor *et al.*, 2017) and emotional charge and this is also associated with work–family conflict (Cortese *et al.*, 2010). Several participants identified a desire to achieve better work–life balance as reasons to leave health visiting.

Bronfenbrenner’s exosystem represents those aspects of the setting that affect new HVs but do not involve them as an active participant. Responses that relate to leaving the profession dominate this category. Reasons for leaving included opportunities to further their career in another role, for increased salary, perceptions about job resourcing in the profession and multi-agency relationships. The impact of market forces is apparent here and the size of this influence is clear from the quantitative findings that show a significant association between leaving the profession and moving to higher paid role, reiterating that dissatisfaction with low pay is a variable associated with intention to leave nursing (Collins *et al.*, 2000; Homburg *et al.*, 2013). Prior to the Health Visitor Implementation Plan, many students took a drop in salary to become an HV (Lindley *et al.*, 2010), and concern by HVs about salary on qualification is a recognised issue, especially as occupations that are traditionally a springboard to health visiting such as midwifery and senior nurse specialists are often paid more than HVs (Baldwin, 2012; Whittaker *et al.*, 2013). Line managers are key to ensuring that the rewards newly qualified nurses receive are sufficient to compensate for their perception of low pay (Homburg *et al.*, 2013).

Several participants cited the number of vacancies in the service having a detrimental impact on their ability to practise in the way that they would like to. Perceptions that the service was under resourced motivated participants to move to roles where they felt they would have more flexibility and autonomy. Similarly, inability to make a difference in a cash-starved hospital environment and the perception of numerous vacancies, and therefore opportunities, in the service were seen as reasons to join the profession. This suggests that the desire to work in a service with adequate numbers of staff and hence be able to undertake professional practice that is meaningful and effective (Whittaker *et al.*, 2013; Twigg and McCullough, 2014) is a constant motivator for career choices. This could be illustrative of the discrepancy between the perception of applicants to HV courses and the reality of the service.

The values, ideology, practices and policies pertaining to health visiting that align with Bronfenbrenner’s macrosystem are influences outside the determination of the newly qualified HV. Whereas beliefs about the value of the role, of public health and early intervention acted as a draw for participants to enter the profession, as did the opportunity created by the Implementation Plan, the restrictions of the scope of practice, lack of respect, not feeling valued, lack of opportunity to progress and organisational culture were cited as reasons for leaving. Whittaker *et al.* (2013) found that making a difference to families was an important aspect of rewarding and worthwhile work experiences but was an aspiration that was difficult to fulfil in practice. HVs described an incongruence between their ideology of practice and tensions and restraints in their work environment and this was a significant threat to retention. This study suggests that they expect to be able to work with professional autonomy, use their skills and knowledge and connect with families, which reiterates previous research in the area (Whittaker *et al.*, 2017). This insight is important for retention strategies and aligns with the concept of a psychological contract between the HV and their employer (Rousseau, 2001). When expectations are not met, this may be regarded as a breach of that contract and influence decisions to leave (Maben, 2008). Government policy is highly influential in the UK health arena and investment in the health visiting service was successful in attracting over 4000 new HVs into the profession. Equally, lack of continued ring-fenced investment (Ford, 2017) has been instrumental in motivating practitioners to leave.

Bronfenbrenner’s final system is the chronosystem, purporting that environments are not fixed identities but change over time as society adopts new discourses. This is particularly relevant to this study as the responses of the participants are contextualised within their developing careers. HV role identity is influenced by constant feedback from other health visitors, relationships with inter-professional colleagues and local and national policies (Machin *et al.*, 2012); and in times of flux, it is difficult to maintain identity equilibrium. Identity confusion is a threat to morale and a risk to retention, especially when that identity is not yet fully formed by newly qualified HVs.

The data were collected one year after the conclusion of the Health Visitor Implementation Plan, and most participants were within the first two years of their new roles. Their professional experience was situated within a complex context of NHS transformation and economic reform. The Health and Social Care Act (HM Government, 2012) set out radical changes to the structure of the NHS, which had a major impact on public health services. Services for children and young people aged 0–19 have been commissioned by Local Authority since 2015 and are now offered by a range of providers including non-NHS organisations, community interest companies, social enterprise organisations and general practitioner general practitioner (GP) consortia. In England, there are 2500 fewer GPs than required (The King’s Fund, 2019). This workforce crisis and wider changes to the healthcare landscape have introduced uncertainty around both the future shape of HVs’ roles and job security (Unite the Union, 2016). As alluded in the introduction, the number of HVs leaving their role has increased since the data were taken and the workforce is now back at pre-Intervention Plan size. Nursing literature indicates that newly qualified nurses often have unrealistic expectations of their new role (Higgins and Green, 2011), leading to frustration and demoralisation, potentially linked to short-lived lack of confidence (Maben *et al.*, 2007). For newly qualified nurses, job satisfaction scores dropped at one year after qualification but at two years’ post-qualification their job satisfaction had increased again (Anderson *et al.*, 2009). These nurses often feel that they still need further support and mentoring after their transition or orientation programme has been completed and may leave the organisation in anticipation of greater support elsewhere (Almada *et al.*, 2004). This may reflect unrealistic expectations of their ability, given their relative inexperience (Mooney, 2007). Relating this to the HV context, preparation for transition to HV registration should involve further contextualisation to meet their specific needs.

## Limitations

Using electronic surveys in nursing research offers a time and cost-efficient way of reaching a large number of geographically disparate participants. However, recognised challenges to using this method include low response rates in comparison with other methods of collecting data (Cho *et al.*, 2013). The response rate for this survey was 19% so potentially non-response bias may impact on the reliability and validity of the data, despite strategies used to encourage participation such as careful design of the survey. Reminder emails were sent by the educational programme leads who were in post at the time of the Implementation Plan to attempt to make a personal connection with the participants, as suggested by Cope (2014) and this did yield further participation. The small number of responses from participants who had left health visiting make it difficult to draw conclusions that could be generalised to the wider population but offer some insight into the issues that newly qualified HVs faced at the time of the survey. A more detailed data set from a wider national survey and in-depth interviews would have enhanced the discussion.

## Conclusion and recommendations

The findings indicate that in order to influence recently qualified HVs to remain in the profession, both local and strategic changes may be required. At the point of recruiting nurses or midwives onto HV education programmes, consideration should be given to prior experience of working in the community for more than three years, as these candidates will have clear expectations of working in a community environment and are more likely to remain in the profession. Enhanced community placements in pre-registration nursing and midwifery support potential HVs to identify whether the community environment fits with their work preferences.

When approaching qualification as HVs, students may require extra support with transition to the role, to effectively manage work–life balance and to mediate expectation and reality. Scrutiny of the work environment by service leaders to determine the elements could be enhanced to meet novice HVs’ needs may also encourage retention.

Strategically, the findings indicate that retention may be improved by developing pathways for career progression, including opportunities for increased remuneration and recognition of value within the workforce. Ring fenced reinvestment in the service has the potential to improve work conditions and the attraction of the specialist public health nursing role.
